# Breast Cancer Therapy by Small-Molecule Reactivation of Mutant p53

**DOI:** 10.3390/curroncol32120684

**Published:** 2025-12-03

**Authors:** Simon H. Slight, Salman M. Hyder

**Affiliations:** Department of Pathobiology and Integrative Biomedical Sciences, University of Missouri, Columbia, MO 65211, USA

**Keywords:** p53, PRIMA1, reactivation, breast cancer

## Abstract

Tumor suppressor p53 is essential for maintaining DNA stability and preventing cancer. Under normal conditions, the p53 protein is either degraded or bound to a negative regulator, rendering it inactive, but when DNA damage occurs, p53 is activated, causing cell cycle arrest and allowing time for cellular repair. If, however, DNA damage is too severe, the cell undergoes apoptosis and is eliminated. Mutations in the p53 gene are linked to various types of cancer and are present in 30–40% of human breast cancers, leading to loss of tumor suppressor function and uncontrolled tumor growth. Moreover, in triple-negative breast cancer (TNBC), a particularly deadly form of the disease, the incidence of p53 mutations increases to 70–80%. Many p53 mutations occur in the DNA binding domain of the p53 gene, leading to accumulation of mutant p53 (mtp53) within the cell, and tumor development. Converting mtp53 back to its functional wild-type form (wtp53) is consequently a rational approach to preventing or even reversing tumor growth. Mechanisms of action of tumor suppressor p53 are widely discussed elsewhere; hence, we will focus on our own studies, using small molecule activators of mtp53 to combat breast cancer. We will show that specific small molecules, such as PRIMA-1 (p53 reactivation and induction of mass apoptosis), reactivate mtp53 in hormone-dependent human breast cancer cells. Furthermore, we will demonstrate the effectiveness of PRIMA-1 at arresting xenograft growth in an animal model and go on to show that the PRIMA-1 analog APR-246 effectively restores wtp53 tumor suppressor activity in TNBC cells. A brief overview of current clinical trials aimed at reactivating p53 to treat certain cancers is provided. Finally, we discuss the possible use of naturally occurring compounds, which are generally non-toxic, to reactivate mutant p53 and control TNBC progression.

## Simple Summary

Breast cancer is the most common type of cancer in American women and, after lung cancer, is the deadliest form of the disease. About 30–40% of human breast cancers contain mutations in a gene that under normal situations prevents tumor growth. Termed tumor suppressor p53, this gene produces a protein that in its wild-type (unmutated) form maintains DNA stability and promotes apoptosis (cell death) of cancer cells. Unfortunately, mutant p53 is unable to prevent tumor growth, so efforts are underway to convert inactive mutant p53 into the active form of the protein. Several small molecules have been developed recently, which bind to mutant p53 and restore its anti-tumor activities. These molecules have been tested against a variety of human cancer cells, and in animal models of cancers that are hormone-dependent, as well as against triple-negative breast cancers, which are particularly aggressive and deadly. There is some evidence that certain naturally occurring, plant-derived compounds also have the capacity to convert mutant p53 into its active, anti-cancer form. Studies are ongoing to determine the effectiveness of such compounds, which are generally of low toxicity compared with currently employed chemotherapies and would provide many breast cancer sufferers with an alternative means of fighting the disease.

## 1. Introduction

In its 2024 biennial report on breast cancer, the American Cancer Society estimated that in 2024 approximately 310,720 new cases of invasive breast cancer and 56,500 cases of ductal carcinoma in situ (DCIS) would be diagnosed in the US [1]. According to this report, about 42,250 women would die of breast cancer, making it the second leading cause of cancer death among women, and the leading cause in Black and Hispanic women. The development of breast cancer is influenced by race, genetic factors, lifestyle choices, and environmental exposures; hence, there are many different types of disease (reviewed in [2]). Cancers that are confined to the milk-producing glands and ducts such as lobular carcinoma in situ (LCIS) and DCIS are termed non-invasive, though these forms of cancer can become invasive if left untreated. The wide variety of breast cancers is further complicated by specific molecular portraits and the absence or presence of different hormone receptors (see Cancer Genome Atlas Network [3]. Tumors can be hormone-receptor positive (HR+), reflecting the presence of progesterone and/or estrogen receptors (PR/ER), HER2 positive, where human epidermal growth factor receptor 2 is overexpressed, or triple-negative. Tumors in the latter case lack ER/PR and HER2 receptors and are termed TNBC, a typically aggressive and metastatic form of the disease. The lack of molecular targets in TNBC renders it particularly difficult to treat using chemotherapies commonly employed against hormone-responsive cancers [4].

## 2. Tumor Suppressor p53

**Figure 1:**
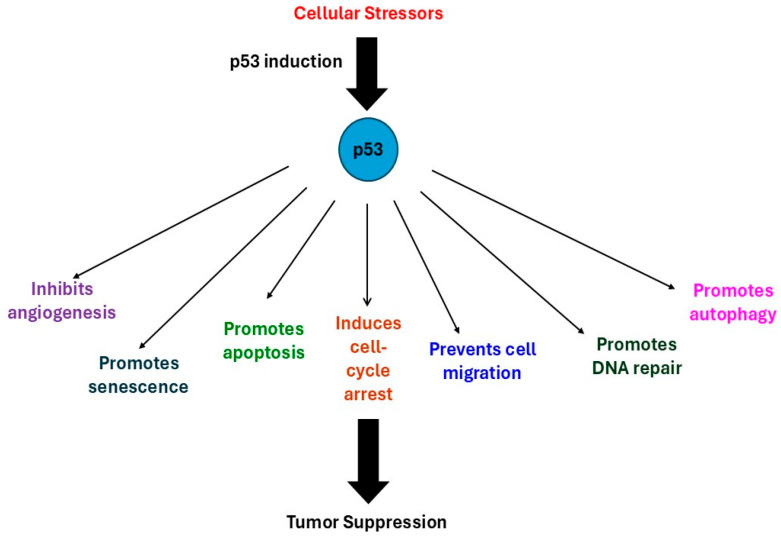
Induction of wtp53 activity by cellular stressors (nutrient depletion, oxidative stress, hypoxia, DNA damage, oncogene expression, ribosomal dysfunction, telomere attrition, viral infections) promotes functions that suppress tumor growth.

A high proportion of breast cancers express a mutant form of tumor suppressor p53 (mtp53), a protein that, under normal, wild-type conditions (wtp53), regulates cellular metabolism and monitors for DNA damage and cellular stress [5,6]. Often referred to as the “guardian of the genome,” p53 regulates cellular homeostasis, promoting cell cycle arrest and apoptosis, and inhibiting VEGF-dependent angiogenesis. p53 prevents tumor growth, metastasis, and potential drug resistance via mechanisms outlined in Figure 1. The self-renewal properties of cancer stem cells (CSCs) are disrupted by p53, as is the epithelial to mesenchymal transition, a sequence of rapid phenotypic changes essential for metastasis [7,8,9]. Most p53 mutations occur within the DNA binding domain, predominantly as missense mutations that increase oncogenic functions, including invasion, metastasis and resistance to apoptosis [3,10]. Mutation “hot-spots” occur at R175H, Y220C, G244C, G245S, R273H and R282W, resulting in changes in transcriptional regulator function that disrupt normal gene expression and promote uncontrolled growth of cancer cells [10]. Mutations in p53 cause reduced activation of tumor suppressor genes, leading to tumorigenesis and the development of aggressive, invasive drug-resistant tumors [11]. The deleterious downstream effects of p53 mutations highlight the importance of developing strategies to target mtp53 and to restore normal tumor suppressor functions.

## 3. Restoration of wtp53 Activity by PRIMA-1

**Figure 2:**
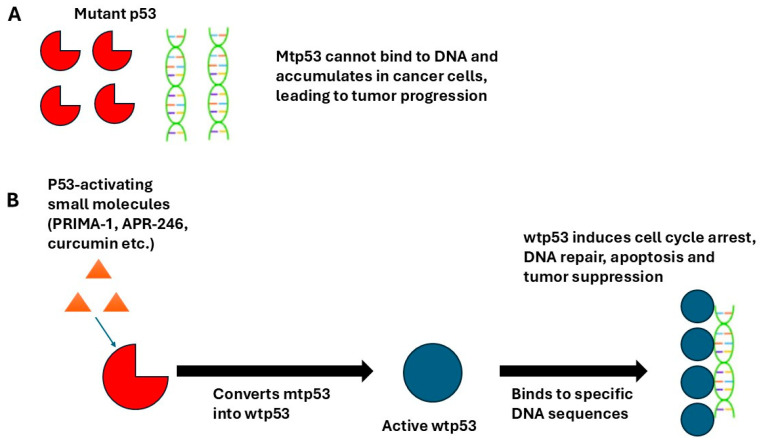
Reactivation of mtp53 to wtp53 by small molecules leads to p53/DNA binding and tumor suppression. (**A**) Inactive mtp53, (**B**) Activation of p53 by small molecules restores wtp53 activity.

Over the years we have undertaken studies to reactivate wtp53 and to inhibit the growth of a variety of human breast cancer cell lines and disrupt the development of tumors. We demonstrated that transfection of the wtp53 gene into hormone-responsive T47-D human breast cancer cells suppressed their growth. In xenograft studies involving estrogen-supplemented nude mice inoculated with wtp53 transfected T47-D cells, tumors grew slowly and quickly regressed [12]. The 2002 discovery [13] that a non-toxic, low-molecular-weight compound, PRIMA-1, binds to DNA in a sequence-specific manner and induces apoptosis of cancer cells (Figure 2), led to us conducting a series of studies with the compound in a variety of human breast cancer cells. PRIMA-1 was shown to reactivate p53 in cells expressing a variety of different mutants, including R175H, G245S, R248W (the most common p53 mutation), R273H and R282W [13].

Initially, we studied how PRIMA-1 affects the expression of vascular endothelial growth factor (VEGF) in several different hormone-responsive human breast cancer cell lines, which differ in their expression of estrogen and progesterone receptors (ER, PR), as well as their wtp53/mtp53 expression and content [14]. We found that VEGF inducibility by progestins is correlated with PR expression and p53 deficiency. Furthermore, activation of mtp53 by PRIMA-1 blocks the stimulation of VEGF expression by progestins in PR-positive p53-deficient cells. In cultured T47-D cells, which contain mtp53, PRIMA-1 suppressed VEGF expression, while inducing expression of estrogen receptor beta (ERβ), indicating conversion of mtp53 to wtp53 and reactivation of p53 activity [12]. Immunofluorescence staining showed that PRIMA-1 restores the wtp53 conformation in cultured human BT-474, HCC-1428 and T47-D breast cancer cells, which normally express the mutant form of the protein [15]. Furthermore, PRIMA-1 significantly reduced the volume of xenograft tumors derived from the three aforementioned human cell lines in estrogen supplemented nude mice, demonstrating a general in vivo inhibition of mtp53-expressing cancer cells, disruption of cell proliferation and attenuation of tumor growth [15].

The in vivo effects of PRIMA-1 on BT-474- and HCC-1428-derived xenografts in nude mice were examined [16]. In these studies, mice were also treated with a monoclonal antibody (2aG4) that targets exposed phosphatidylserine residues on tumor blood vessels and disrupts tumor vasculature. Given singly, both PRIMA-1 and 2aG4 inhibited tumor progression. However, a combination of PRIMA-1 and 2aG4 was significantly more effective than either compound alone; in the BT-474 model approximately 30% of tumors were completely eradicated [16]. Tumor tissues were analyzed immunohistochemically for vascular endothelial growth factor (VEGF) and CD-34, a marker for blood vessels. Both PRIMA-1 and 2aG4 treatment reduced VEGF and CD-34, with a combination of the two eliciting a greater reduction than either agent alone. Tumor blood vessel density and perfusion were likewise diminished, indicating that especially in combination, PRIMA-1 and 2aG4 antibody disrupt angiogenesis and effectively oppose tumor growth [16].

Using an inverse-docking approach, we sought to identify alternative molecular targets for PRIMA-1. This led to the identification of oxidosqualene cyclase (OSC), a key enzyme in the biosynthesis of cholesterol as a potential target [17]. RO 48-8071 (RO) inhibits OSC and studies in our laboratory show that, like PRIMA-1, RO suppressed the in vitro viability of both BT-474 and T-47D human breast cancer cells, while having no effect on normal mammary cells. Furthermore, RO promoted the binding of mtp53 to DNA, in much the same fashion as PRIMA-1 [17].

## 4. Triple-Negative Breast Cancer

Triple-negative breast cancer (TNBC) is an especially aggressive disease subtype that accounts for 10–20% of all breast cancer cases worldwide [18,19]. TNBC lacks ER and PR, as well as HER-2/neu, which are common targets for chemotherapeutic agents, making these cancers difficult to treat. As a result, TNBCs, which are rich in stem cells, tend to metastasize, leading to poor patient outcomes. Inactive p53 tumor suppressor protein occurs in approximately 80% of TNBCs, leading to rapid tumor growth and metastasis. Therefore, it is crucial to develop novel treatments for this particularly deadly form of cancer. As outlined in our previous review [20], the high prevalence of mtp53 in TNBC and its significant impact on disease progression underscore the importance of reactivating its functional wild-type form in TNBC patients.

## 5. APR-246 Studies

A more effective form of PRIMA-1 is generated by the addition of a methyl group, to produce PRIMA-1Met (termed APR-246 and more recently epranetapopt). APR-246 is more soluble than PRIMA-1. We previously described in some detail the mechanisms through which PRIMA-1 and APR-246 restore p53 activity [20] and to do so here would be beyond the scope of the present manuscript. It is known that APR-246 covalently binds to cysteine residues within the DNA-binding domain of mtp53, furthermore, it reactivates mutations R175H and R273H, thereby restoring the wild-type conformation and enhancing its capacity to block the cell cycle and induce apoptosis [21]. The cytotoxicity of APR-246 is dose- and time-dependent and depends on several factors. These include cell confluence, hypoxia, cell-cycle progression, and specific cell line sensitivities [22,23]. APR-246 acts specifically against cells that contain mtp53, as evidenced by reduced cytotoxic effects in p53 null cells and cells expressing wtp53 [22,23]. We have conducted extensive studies, both in vitro and in vivo, to ascertain its effectiveness against hormone-responsive breast cancer and TNBC. Cell viability of BT-474 and T47-D human breast cancer cells, both of which express mtp53, was reduced by APR-246 [24]. APR-246 increased DNA binding of BT-474 cells, indicating conversion of mtp53 into wtp53, and induced BT-474 cellular apoptosis. Studies were conducted in nude mice bearing BT-474 or T47-D tumor xenografts. Animals were treated with either APR-246 or the monoclonal antibody 2aG4, or a combination of both. Combination treatment more effectively reduced the proliferation of cancer cells, induced their apoptosis and reduced tumor growth more than either agent alone [24]. Animals treated with doses of APR-246 up to 100 mg/kg/every other day maintained body weight and exhibited no behavioral changes, whether the drug was administered intravenously, intraperitoneally, or by intra-tumoral injection [24,25]. Thus, we are confident that APR-246 is non-toxic and may be used therapeutically against a variety of breast cancer types, offering a safe alternative to current highly toxic, non-targeted chemotherapy regimens.

APR-246 was the first mtp53-reactivating small molecule drug to advance into late-stage clinical trials, earning its designation as a first-in-class drug. Initially, APR-246 underwent phase I and II clinical trials targeting malignancies of the blood and prostate [26]. These trials measured the maximum tolerated dose of APR-246, and evaluated its overall safety, dose-limiting toxicity, and pharmacokinetics. A total of 22 patients, aged between 55 and 81 years, participated in the study. Eligibility required a life expectancy greater than two months. While p53 status was not a selection criterion, approximately 25% of participants exhibited the mtp53 conformation. APR-246 was administered intravenously for four consecutive days, leading to the determination of the MTD as 60 mg/kg. The drug demonstrated a favorable safety profile, with common side effects such as fatigue, dizziness, headache, and confusion, though less severe than those observed with many standard chemotherapeutic agents. Additionally, favorable pharmacokinetics were observed, characterized by minimal interindividual variations independent of dose or time.

Tumor cells in many patients exhibited signs of cell-cycle arrest, heightened apoptosis, and upregulation of p53 target genes. For example, in one case involving a patient with acute myeloid leukemia and a core p53 mutation, the blast percentage decreased from 46% to 26%. Of the six patients presenting with circulating malignant cells, four demonstrated cell-cycle changes, including complete arrest. Furthermore, an upregulation of p53 downstream targets such as Bax, PUMA, and NOXA, as well as the senescence marker DcR2, was also observed [26].

The initial success of APR-246 in clinical trials suggests that combining this small-molecule drug with other agents could prove to be an effective strategy for treating TNBCs. Having assessed the efficacy of APR-246 against hormone-responsive breast cancers, we conducted studies to examine its effectiveness against TNBC, again both alone and in conjunction with the tumor blood vessel disrupting antibody 2aG4 [25,27]. MDA-MB-231 and MDA-MB-435 TNBC cells were exposed to APR-246 and its effects on aldehyde dehydrogenase (ALDH) activity, mammosphere formation and cell migration assessed. Increased ALDH activity and the ability to form mammospheres are characteristic biomarkers of stem-cell-like cells within tumors and are major hallmarks of metastatic TNBC. All three parameters were disrupted in vitro by APR-246 [27]. By blocking blood vessel function and reducing angiogenesis, both critical for tumor progression, 2aG4 contributes to tumor cell death. In nude mice inoculated with MDA-MB-435 TNBC cells, both APR-246 and 2aG4 significantly reduced the average number of metastatic colonies in lungs [27]. A combination of APR-246 and 2aG4 was even more effective, virtually wiping out lung metastasis compared with untreated controls. Furthermore, although the use of a single agent alone only lowered the incidence of lung metastasis slightly within treatment groups, combination treatment reduced the number of mice exhibiting metastatic lung tumors by almost half. When used together, APR-246 and 2aG4 typically cause ischemia, hemorrhagic necrosis, and eventual cell death in both hormone-dependent breast cancer and TNBC. Our studies demonstrate that treatment of tumor-bearing animal models with APR-246 and 2aG4 is non-toxic, effectively suppresses tumor growth and, in some cases, achieves complete tumor eradication. Additionally, by reducing tumor blood vessel density, the combination treatment may help prevent metastasis and the development of secondary malignancies.

## 6. Other Small Molecule Activators of mtp53

Concomitant with early studies on PRIMA-1, several alternative small molecules were identified that restored p53 activity to mtp53. These include CP-31398, STIMA-1 and MIRA-1 (together with its analogues MIRA-2 and MIRA-3) [28,29]. These compounds reactivate p53 functionality and promote expression of p53 target genes and apoptosis. STIMA-1 was shown to target tumor cells that specifically express the R175H and R273H mutations, though in vivo studies against human tumor xenografts were hampered by its low solubility [29].Following the success of PRIMA-1 and, subsequently, APR-246, to reactivate mtp53, the anti-tumor effects of other small molecules that bind to mtp53 have been examined. The third-generation thiosemicarbazone COTI-2 possesses antitumor activity against various cancer types, including TNBC cell lines such as MDA-MB-231, both in vitro and in vivo in animal xenograft models [30]. COTI-2 exhibits greater activity and tolerance in animal models than traditional chemotherapies and appears to reactivate mtp53 in much the same way as APR-246, restoring its DNA-binding ability and its capacity to induce expression of target genes, though research suggests that COTI-2 also has p53-independent anticancer effects, including activation of tumor suppressor AMPK and inactivation of the mTOR oncogene [31,32]. NSC2287, Reactivation of p53 Induction of Tumor Cell Apoptosis (RITA), binds to mtp53, restoring its transcriptional functions and inducing apoptosis [33]. RITA functions independently of p53 status, inducing apoptosis in cancer cells that express either mtp53 or wtp53, or even p53-null cells, showing its ability to function both dependently and independently of p53 status [34]. Activation of the JNK/p38 pathway and down-modulation of anti-apoptotic Bcl-2 are two mechanisms through which RITA stimulates p53-independent pathways that lead to DNA damage and apoptosis. However, despite its promising anticancer effects, RITA caused pulmonary edema in multiple animal models, leading to the termination of its development program [35]. Current research focuses on creating less toxic analogues to potentially make RITA safe for clinical use.

Recently, investigators identified two new compounds, rezatapopt (PC14586) and PC14374, selected from a library of small molecules that reactivate p53-Y220C [36,37]. The investigators focused their studies on rezatapopt and an assessment of its capacity to restore tumor suppressor function of p53 mutants with the specific Y220C substitution. Rezatapopt restored wtp53 activity, leading to upregulation of p53 target genes and repression of genes involved in cell-cycle progression [36]. Furthermore, oral administration of rezatapopt in two mouse xenograft models (NUGC-3 and T3M-4) caused a rapid restoration of wtp53 function, with subsequent activation of downstream targets and inhibition of tumor development. The safety and efficacy of rezatapopt in animal models led to the compound being evaluated in a Phase I clinical trial (PYNNACLE), in which patients with a variety of different tumors, already heavily pretreated with conventional chemotherapy regimens, were administered rezatapopt [38]. Early results suggest that rezatapopt is well tolerated and shows effectiveness against solid tumors expressing the Y220C mutation, including in a patient with TNBC [39], though it should be noted that the specific Y220C mutation is relatively uncommon and only occurs in about 2% of all tumors [40]. An ongoing Phase II clinical trial (available at: https://www.pynnaclestudy.com/) will further evaluate its potential for use in patients with this specific p53 mutation.

It has been shown that breast cancer mortality is significantly reduced in women exposed to high levels of inorganic arsenic in drinking water [41]. Furthermore, arsenic trioxide (ATO), a small molecule drug already in clinical use for treating acute promyelocytic leukemia (APL), shows promise as a potential treatment for TNBC [42]. ATO reactivates mtp53, thereby restoring wtp53 functionality. ATO inhibits proliferation in mtp53 cell lines more effectively than in cell lines containing wtp53 and was found to be more potent against triple-negative than non-triple-negative cell lines. A combination of ATO with commonly used chemotherapeutic drugs such as doxorubicin and docetaxel produced synergistic effects against mtp53 containing cells. ATO induced apoptosis in a variety of breast cancer cell lines and upregulated expression of wtp53 target genes. Clinical trials are ongoing to ascertain the effects of ATO against a variety of mtp53 cancers, including ovarian and endometrial cancer, and leukemias. The toxicological effects of ATO are well-established in patients undergoing therapy, with manageable adverse consequences, including fever, mild hepatotoxicity, infections and edema [43]. Based on these studies, there is justification for clinical studies to examine the effectiveness of ATO against TNBC.

## 7. Restoration of wtp53 Activity by Natural Compounds

There is increasing evidence that naturally occurring, plant-derived compounds possess important antitumor properties. Phytochemicals such as luteolin, apigenin and curcumin have been shown to disrupt proliferation of cancer cells and arrest tumor development. Curcumin upregulates p53, increases its half-life and promotes apoptosis in MDA-MB-231 TNBC cells. A discussion of its complex mechanisms of action can be found elsewhere [44], but it has been shown in human pancreatic cancer cells that curcumin restores wild-type activity at the p53Y220C mutation site and induces apoptosis of cancer cells [45,46]. A curcumin analog, HO-3867, has been shown to preferentially bind to mtp53 and convert it into active wtp53 in human cancers that express mtp53 [47], including T47D and MDA-MB-468 (TNBC) human breast cancer cells. An in vivo luciferase-based reporter transcription assay, using the same breast cancer cell lines provided further confirmation that HO-3867 induced transcription by converting mtp53 to wtp53. HO-3867 induced apoptosis of mtp53-bearing breast cancer cells and reduced the volume of tumors in an animal xenograft model. The authors thus concluded that HO-3867 transformed and restored mtp53 activity by changing its conformation [47].

We studied the effects of liquiritigenin (LQ), a flavonoid derived from licorice root, on hormone-dependent breast cancer and more recently on TNBC. LQ, an ERβ agonist, reduced the in vitro viability of MCF-7 and BT-474 human breast cancer cells [48]. When used in conjunction with RO, LQ was even more effective. Both compounds inhibited tumor growth in an in vivo mouse xenograft model and a combination of RO and LQ was significantly more effective than either compound alone. Similar results were obtained when the effects of RO and LQ were examined in TNBC cells and in a xenograft model derived from MDA-MB-231 TNBC cells [49]. Both LQ and RO induced expression of ERβ and reduced expression of angiogenesis markers in tumor xenograft cells. Hence, we attributed the antitumor effects of LQ to its ability to both induce ERβ expression and act as an ERβ agonist. However, it was recently shown by computational analysis and cell-based studies that as with curcumin, LQ and other flavonoids exert anti-tumor effects by restoring the wild-type conformation of p53Y220C mutants [50], suggesting that the actions of these naturally occurring compounds are multi-faceted. It is clear therefore that curcumin and other phytochemicals warrant further investigation for their ability to reactivate mtp53 since these naturally occurring and typically non-toxic substances could offer safe treatment options for patients with few effective alternatives. Optimizing such compounds could lead to the development of potent, yet non-toxic, analogues, further expanding the arsenal of therapeutic agents for TNBC and other cancers.

## 8. Conclusions

Efforts to target mtp53 and restore normal p53 tumor suppressor activity have intensified in recent years. Compounds such as APR-246, COTI-2, rezatapopt, and, most recently, ATO, are examples of small molecules that show promise in clinical trials. Drug design, based on rational structure–activity relationships, together with emerging gene- and cell-based technologies, offer exciting opportunities for treatment. The effectiveness of small molecules against a variety of cancers, including those of the breast, is being examined. We initially showed, in animal models, that PRIMA-1 and APR-246 effectively inhibit the development of tumors in xenografts derived from human breast cancer cells; their activity was bolstered by the concomitant administration of 2aG4, an antibody that targets tumor blood vessels. Most TNBC cases are associated with mtp53 mutations; consequently, new therapies targeting mtp53 are especially critical for patients with this aggressive type of cancer, which currently lacks specific targeted treatments. APR-246, both alone and in combination with 2aG4, reduces significantly the metastasis of TNBC cells to the lungs, raising the possibility that such treatments may represent a significant breakthrough in the treatment of metastatic breast cancer. Naturally occurring phytochemicals possess anti-tumor properties. Studies show that curcumin restores the wild-type activity of human cancer cells expressing the p53Y220C mutation, and the curcumin analog HO-3867 converts mtp53 to its active form in TNBC cells. As progress is made in the therapeutic restoration of wtp53 activity as a means of combating cancer, there must be an awareness that over-activation of p53 might also be deleterious to the patient and could ultimately cause more harm than good. Since p53 promotes cell cycle arrest and apoptosis, there is the potential for p53 hyperactivation to damage or destroy normal cells, leading to strokes, cardiac arrest and cell death in degenerative diseases such as arthritis and multiple sclerosis. However, based on our extensive studies, compounds that reactivate mtp53 are well tolerated by experimental animals and there are few if any signs of toxicity at the concentrations administered.

## Data Availability

No new data were created or analyzed in this study.
